# Transcription factor 4 and its association with psychiatric disorders

**DOI:** 10.1038/s41398-020-01138-0

**Published:** 2021-01-05

**Authors:** José R. Teixeira, Ryan A. Szeto, Vinicius M. A. Carvalho, Alysson R. Muotri, Fabio Papes

**Affiliations:** 1 Department of Genetics, Evolution, Microbiology and Immunology, Institute of Biology, University of Campinas, Campinas, São Paulo Brazil; 2 Department of Pediatrics/Rady Children’s Hospital, School of Medicine, University of California San Diego, La Jolla, CA USA; 3 Department of Cellular & Molecular Medicine, School of Medicine, University of California San Diego, La Jolla, CA USA; 4 Kavli Institute for Brain and Mind, University of California San Diego, La Jolla, CA USA; 5 Center for Academic Research and Training in Anthropogeny (CARTA), University of California San Diego, La Jolla, CA USA

**Keywords:** Molecular neuroscience, Autism spectrum disorders, Schizophrenia

## Abstract

The human transcription factor 4 gene (*TCF4*) encodes a helix–loop–helix transcription factor widely expressed throughout the body and during neural development. Mutations in *TCF4* cause a devastating autism spectrum disorder known as Pitt–Hopkins syndrome, characterized by a range of aberrant phenotypes including severe intellectual disability, absence of speech, delayed cognitive and motor development, and dysmorphic features. Moreover, polymorphisms in *TCF4* have been associated with schizophrenia and other psychiatric and neurological conditions. Details about how *TCF4* genetic variants are linked to these diseases and the role of TCF4 during neural development are only now beginning to emerge. Here, we provide a comprehensive review of the functions of *TCF4* and its protein products at both the cellular and organismic levels, as well as a description of pathophysiological mechanisms associated with this gene.

## A primer on the human transcription factor 4

Transcription factor 4 (TCF4) is a member of the helix–loop–helix (HLH) family of proteins expressed in several cell types and tissues throughout the body^1^. Mutations in the *TCF4* gene are known to cause the autistic condition known as Pitt–Hopkins syndrome (PTHS)^2–4^. Moreover, genome-wide association studies (GWAS) identified *TCF4* polymorphisms linked with schizophrenia (SCZ) and other psychiatric conditions^5–10^, as well as non-neurological genetic diseases^11–13^. Importantly, *TCF4* is abundantly expressed during neural development^1,14,15^, which likely reflects its relevance for the nervous system. In this review, we describe the known functions of TCF4 and the pathological consequences of *TCF4* genetic variants linked to psychiatric disorders.

*TCF4* is the gene’s official symbol (HUGO Gene Nomenclature Committee), but it is also frequently referred to as *E2-2*, immunoglobulin transcription factor 2(*ITF2*), or *SEF2-1*. These different names stem from the distinct contexts in which the gene was originally described. E2-2 was the name given to a cDNA from a B-cell library, which encoded a protein—named ITF2—that interacted with enhancers in the immunoglobulin heavy and light chain loci^16^. An independent study purified two proteins from helper T-cell extracts and showed that they bind the murine leukemia virus SL3-3 enhancer; the proteins, named SL3-3 enhancer factor 2-1A and -1B (SEF2-1A/B), were later identified as ITF2 isoforms^17^. It is important to mention that TCF4 should not be confused with the immune regulator transcription factor 7-like 2 (TCF7L2), a member of the TCF/LEF family of transcription factors also referred to as T-cell factor 4 (TCF4), which therefore confusingly shares the same gene symbol.

TCF4 binds to DNA as either homo or heterodimers^15,18–24^, a phenomenon shown to increase HLH DNA–binding specificities and transcriptional control capacity^25^. The HLH family is characterized by the presence of a highly conserved dimerization domain formed by two amphipathic α-helices separated by a loop, hence the name “Helix–Loop–Helix”^25^. HLH transcription factors also carry a highly conserved group of basic residues at the N-terminus of the first helix, which is critical for DNA binding, being the reason why they are sometimes called basic helix–loop–helix (bHLH) proteins^25^.

## Association between *TCF4* polymorphic variants and psychiatric diseases

GWAS identified *TCF4* polymorphisms associated with SCZ^5–7^, bipolar disorder^7,8^, post-traumatic stress disorder^10^, and major depression disorder^9^. *TCF4* polymorphisms have also been associated with the non-neurological diseases primary sclerosing cholangitis^13^ and Fuchs’ endothelial corneal dystrophy (FECD)^11,12^. Most *TCF4* polymorphisms identified by GWAS were found in non-coding regions, and it is mostly unknown if and by which mechanisms these polymorphisms are causally linked with disease onset. An exception is FECD, for which the molecular pathology has been determined: most patients with FECD carry an expansion of the trinucleotide repeat (CTG)_n_ in a *TCF4* gene intron, leading to foci of condensed poly(CUG) RNAs complexed with splicing factor MBNL1 in the nucleus; sequestration of MBNL1 then results in erroneous splicing of its target mRNAs^12,26,27^.

Even though the causal relationship between *TCF4* genetic variants and SCZ has not been demonstrated, certain polymorphisms are indeed associated with SCZ and have been studied regarding their correlation with the disease’s endophenotypes (see ref. ^28^ for an extensive review of aberrant phenotypes in patients with SCZ and their relationship with *TCF4* polymorphisms). Different risk alleles have been associated with reduced sensorimotor gating as measured by pre-pulse inhibition^29^, modulation of sensory gating by smoking behavior as measured by P50 suppression of the auditory evoked potential^29^, poor verbal fluency performance^30^, and lower reasoning and problem-solving performance^31^. Curiously, some *TCF4* risk variants in patients with SCZ have been associated with enhanced performance in word recognition^32^ and better performance in several attention-related tasks (but worse performance in unaffected individuals)^33^.

Experiments using neurons differentiated from patient-derived induced pluripotent stem cells showed that *TCF4* expression was elevated in samples from patients with SCZ as compared to unaffected individuals^34^. Moreover, the association of increased *TCF4* expression with SCZ is supported by *Tcf4* overexpression in transgenic mice, which results in profound deficits in fear memory^35^ and sensorimotor gating^36^.

Interestingly, there are a few rare *TCF4* coding variants detected in sporadic SCZ cases^37,38^, but most of them are localized outside known functional domains. The exceptions are a F211L variant located in an activation domain (AD3) and a P156T variant located in a nuclear localization signal (NLS-1) domain^37,38^. It seems that the coding variants in sporadic SCZ lead to higher activity-dependent TCF4-mediated transcription when compared to the wild-type variants, in reporter assays conducted with rat primary neurons^39^. However, such effect is marginal, suggesting that any possible impact of SCZ coding variants on TCF4 function would be modest.

## Monogenic determination of Pitt–Hopkins syndrome by *TCF4* mutations

In 2007, three studies independently identified mutations in *TCF4* as the genetic cause of PTHS^2–4^. Until 2016, around 300 PTHS cases had been confirmed around the world^40^, although detailed molecular data on the types of *TCF4* mutations carried by these patients were retrieved for approximately 150 individuals only^41^. Irrespective of the numbers reported in the literature, the total case count is certainly higher by now, not only because the disease is caused by de novo mutations, which are expected to arise at a steady rate, but also because some cases are simply not diagnosed or reported, especially in developing countries. PTHS is expected to be equally prevalent worldwide and one study estimated that the prevalence of PTHS caused by chromosomal deletions is 1/34,000–1/41,000^42^. In contrast, the First International Consensus Statement on Diagnosis and Management in PTHS estimated the prevalence as 1/225,000–1/300,000 based on individuals with PTHS in the United Kingdom and the Netherlands^41^.

In recent years, compiled data better delineated the disease’s phenotype and natural history, and helped create clinical diagnosis and treatment protocols^40,41,43–46^. It is now clear that the PTHS phenotypes are highly variable among individuals, but some aberrant phenotypes are found in most patients, including a very peculiar set of dysmorphic facial features combined with intellectual disability, which led Drs David Pitt and Ian Hopkins to first recognize it as a separate medical entity^47^. The characteristic PTHS facial *gestalt* is present in approximately 89% of patients, consisting of a broad beak-shaped nose with flaring nostrils, wide mouth with a bow-shaped protruding upper lip and fleshy lower lip, spaced teeth, ears with thick helices, bitemporal narrowing, enophthalmia, thin eyebrows in the lateral portion, and full cheeks. As individuals age, facial features become more obvious and prognathism may appear. A certain percentage of individuals also display other dysmorphic features, such as single transverse palmar crease (60% of cases), digit anomalies (53%), such as syndactyly or polydactyly, persistent fetal pads (45%), short stature (38%), urogenital malformations (32%), scoliosis (20%), as well as a tendency to exhibit smaller-than-normal head circumference (~59% of cases)^40,41,43–46^.

All individuals with PTHS have intellectual disability and disturbed sensorimotor gating, speech delay, mild-to-severe motor delay, as well as generalized hypotonia. About 78% of patients frequently engage in stereotypical and intense repetitive movements or behaviors, which put individuals with PTHS within the autistic spectrum if considered in combination with deficits in communication and social interaction beyond what would be expected for individuals with low cognitive levels, motor and speech delay^48^.

Approximately half of patients with PTHS display abnormal breathing patterns, which start on average at 6 years of age and typically consist of tachypnea followed by apnea that lasts for a few minutes, occurring from several times in 1 hour to a few times in 1 year^40,41,43–46^. Periods of tachypnea and apnea may occur independently or be induced by arousal, stress, or anxiety, and periods of apnea may be followed by cyanosis^41^.

About a third of all patients develop epileptic seizures, which may start in the first year of life or even in early adulthood. In a few individuals, abnormal breathing precedes seizures, but the former is not due to epileptic activity, because EEG may be normal in children with respiratory anomalies. Furthermore, some brain abnormalities have been identified by magnetic resonance imaging, such as small or absent *corpus callosum*, large ventricles, and abnormally shaped posterior cranial fossa^40,41,43–45^.

Gastroenterological manifestations are common in individuals with PTHS and include constipation (70% of cases), reflux (35%), and eructation (29%). Other functional deficits are myopia (52%), hyperopia (22%), strabismus (44%), nystagmus (14%), sleep disturbances (18%), and deafness (10%)^40,41,43–46^. Respiratory and urinary tract infections occur in one third of individuals, mainly during childhood. Notably, immunological changes—represented by low levels of IgA, IgG, and IgM—have been sporadically reported, but their relationship with recurrence of infections is still uncertain^41^.

## Transcription factor 4: an elusive molecular player

HLH proteins are grouped into different classes based on the types of dimers they form, patterns of expression, and specificity of DNA binding^25^. TCF4 is a member of the class I HLH group (also named E-proteins)^49^, because it is widely expressed and binds as homo or heterodimers to the consensus sequence CANNTG (“Ephrussi box” or E-box)^24^.

### TCF4 protein domains

**Fig. 1:**
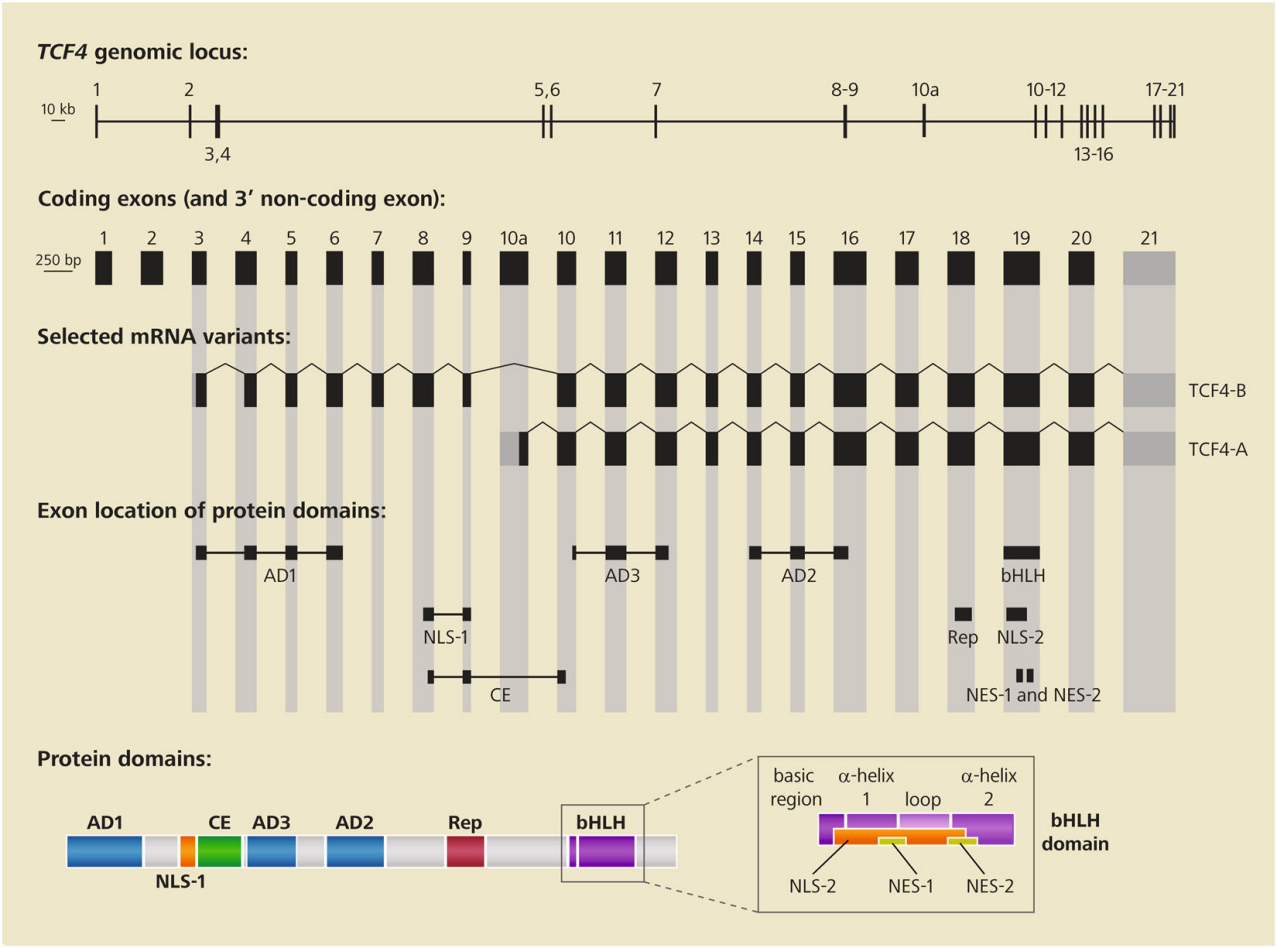
Schematic representation of the *TCF4* genomic locus. The genomic locus is located on chromosome 18 (top line; exon sizes and distances between exons shown to scale), coding exons (middle lines; exon sizes, but not distances, shown to scale), and TCF4 protein domain structure (bottom line; domain sizes not to scale). The last exon on the right, displayed in gray, is non-coding. Only transcript variants coding for isoforms TCF4-B and TCF4-A are shown, which are transcribed from alternative promoters starting at exons 3 and 10, respectively. AD activation domain, NLS nuclear localization signal, NES nuclear export signal, bHLH basic helix–loop–helix DNA-binding domain, CE conserved element, Rep repression domain. In the bottom line, the protein motifs inside the bHLH domain are shown in detail in the enlarged inset. Based on refs. ^22,61^.

Besides the C-terminal HLH domain responsible for dimerization and DNA binding, TCF4 and other E-proteins have N-terminal domains responsible for transcriptional regulation. Full length E-proteins usually contain three conserved activation domains (AD1, AD2, and AD3; Fig. 1) that are able to modulate transcription and, depending on cell type, can independently or cooperatively regulate expression of target genes^50–55^. AD1 is able to bind transcriptional co-activators p300/CBP and STAGA, as well as co-repressor ETO, through the PCET motif (**p**300/**C**BP and **E**TO **T**arget)^53,54,56–60^. AD2 also binds to p300/CBP^53,55^, but there is no evidence that it can recruit ETO or any other transcriptional co-repressor. Transcriptional co-activators p300/CBP and STAGA remodel chromatin through their intrinsic histone acetyltransferase activity and recruitment of basal transcriptional machinery. Transcriptional co-repressor ETO, on the other hand, promotes DNA condensation through recruitment of histone deacetylases. Therefore, p300/CBP, STAGA, and ETO compete for binding to AD1 and, as a consequence, TCF4 may either activate or repress target genes. The third activation domain, AD3, is located between AD1 and AD2 (Fig. 1) and has been shown to directly interact with the TAF4 subunit of general transcription factor II D, part of the basic transcriptional machinery, enhancing the formation of the RNA polymerase II preinitiation complex at target genes^61^. Despite data obtained from deletion studies^62^, it remains to be further explored how AD3 participates in the transcriptional regulation exerted by TCF4, particularly in the nervous system.

E-proteins also contain two intramolecular regulatory domains. The first, termed “conserved element” (CE), is located between AD1 and AD3 (Fig. 1) and is able to repress AD1 activity^63^. The second, termed “repression domain” (Rep), falls between AD2 and HLH (Fig. 1) and was shown to repress the activity of both AD1 and AD2^64^. CE and Rep intramolecular regulatory domains probably act by preventing the recruitment of transcriptional co-factors. Therefore, intramolecular regulatory events repress AD1-mediated transcriptional activation or repression if the genomic context is inclined to either recruitment of transcriptional co-activators or co-repressors, respectively.

Another possible mode of intramolecular regulation seems to occur in TCF4 through a 4-residue sequence—Arg-Ser-Arg-Ser (RSRS)—located between the Rep and HLH domains. Its presence decreases transcriptional activity^65^, but such effect is not observed in all cell types^22^.

Besides having domains directly involved in transcriptional regulation, mammalian E-proteins contain conserved NLSs (NLS-1 and NLS-2; Fig. 1) and nuclear export signals (NES-1 and NES-2; Fig. 1). NLS-1 overlaps with the CE domain at the N-terminal region and NLS-2 overlaps with NES-1 and NES-2 in the HLH domain at the C-terminal region^66^. Further work is required to understand how these domains interact with each other and with other proteins to regulate TCF4 activity in vivo.

### *TCF4* gene structure and transcript diversity

The human *TCF4* gene is located on chromosome 18, spanning approximately 442 kb in chromosomal region 18q21.2, harboring 41 exons, of which 20 are alternative 5’ exons, 20 are internal coding exons, and 1 is the 3’ terminal non-coding exon. Alternative transcription start sites, which are found upstream of internal exons 1, 3, 4, 5, 7, 8, and 10, are responsible for the generation of at least 18 N-terminally distinct protein isoforms, termed TCF4-A through TCF4-R (summarized in Fig. 1)^22^. It should be noted that transcript diversity is even higher, due to alternative splicing of internal exons.

Because of *TCF4*’s structure and regulation, different isoforms may or may not contain AD1, NLS-1, CE, or RSRS sequences. As AD1 is encoded in exons 3–6 (Fig. 1), with the PCET motif coded for only by exon 3, the longer isoforms (TCF4-B, J, K, and L) are the only ones containing a complete AD1 domain with the PCET motif. Other isoforms, such as TCF4-C, contain only parts of AD1 or lack it entirely^22^. Furthermore, as NLS-1 and CE are encoded in exons 8–9 and 8–10, respectively, splicing out the cassette exons 8 and 9 generates “Δ isoforms,” different from their “complete” cognates by the lack of NLS-1 and CE. Moreover, the presence of two alternative donor splice sites in exon 18 allows the inclusion or exclusion of a 12-nucleotide segment encoding the RSRS sequence, present in positive (+) isoforms but absent in negative (–) isoforms. As all TCF4 transcripts have exons 10–20, all isoforms contain AD3 (exons 10–12), AD2 (exons 14–16), Rep (exon 18), and HLH, NLS-2, NES-1, and NES-2 (exon 19)^22^.

### Spatial pattern of *TCF4* expression

*TCF4* spatial pattern of expression is often described as ubiquitous, being detected in multiple organs throughout development^22,67^. However, some *TCF4* transcripts are only detected in a few tissues, while others are widely expressed (see ref. ^22^ for a detailed description of patterns of expression for each transcript). Moreover, expression levels vary between tissues, as certain *TCF4* transcripts may be more or less abundant than others in a particular tissue^22,67^. Curiously, *TCF4* transcripts encoding (+) and (–) isoforms are equally abundant, whereas transcripts encoding Δ isoforms are less abundant than those encoding the complete cognate isoforms. Detailed RT-PCR analysis of the expression of a wide range of different *TCF4* transcripts revealed that, although a stark majority of transcripts display expression in the brain, those coding for five isoforms (TCF4-J, TCF4-K, TCF4-L, TCF4-M, and TCF4-N) are predominantly expressed in the testis and absent in the brain^22^.

## Spectrum of *TCF4* mutations in patients with Pitt–Hopkins syndrome

### *TCF4* genetic variants carried by children with PTHS

The list of *TCF4* mutations found in the hundreds of patients with PTHS described so far include missense (~15% of cases), nonsense (~15%), and splice-site (~10%) point mutations, as well as small insertions or deletions (indels) resulting in frameshift (~30%), and translocations and large deletions encompassing *TCF4* partially or entirely (~30%)^23,41,43–46^.

Depending on the location and type of mutation, the *TCF4* gene and its protein products are differently affected. Most *TCF4* missense mutations in PTHS are located in exon 19, which encodes the HLH domain, but there are cases where the missense mutation is in exons 15 or 18, which encode part of AD2 and Rep, respectively^23^. Importantly, these mutations affect all isoforms. Most nonsense and frameshift mutations and all splice-site mutations affect all isoforms, but there are cases where nonsense and frameshift mutations are located in exons 8–9 and upstream of exons 10a–c, sparing the Δ and the shorter isoforms, respectively^23^. Some translocations and deletions encompass only initial exons (1 through 4) or intermediate exons (5 through 9), sparing intermediate and shorter isoforms, respectively^23^.

The impact of the structural diversity and expression dynamics of *TCF4* on physiology remains a mystery, but some have suggested that the different types of mutation in individuals with PTHS could affect the encoded protein differently and therefore underlie the phenotypic variability observed among patients^68^. Bedeschi et al.^68^ raised the possibility of classifying patients with *TCF4* mutations into three groups: (I) non-syndromic patients with mild intellectual disability or syndromic patients with mild intellectual disability but presenting only a few of the PTHS dysmorphic features; (II) syndromic patients with mild-to-severe intellectual disability but without seizures and with milder facial features; and (III) syndromic patients with severe intellectual disability presenting the characteristic PTHS facial *gestalt*. The authors hypothesized that such classification correlates with TCF4 isoforms affected by the mutations carried by the patients, as individuals in groups I, II, and III usually carry mutations that affect only AD1 (present in the longer isoforms), only NLS-1/CE (present in the longer and intermediate isoforms, but not in their Δ cognates), or AD2/Rep/HLH domains (present in all isoforms), respectively^68^.

Interestingly, in 2016, two different cases were reported of family members with mild intellectual disability carrying a hereditary *TCF4* mutation^69,70^. Both cases strongly support the genotype/phenotype paradigm, showing that certain *TCF4* mutations result in mild pathophysiology, not incompatible with reproduction. On the other hand, there are a few described cases of mild PTHS phenotype where a frameshift mutation located in exon 20 elongates the coding region to the terminal non-coding exon 21, not directly affecting any known domains but affecting all isoforms^71–73^. One hypothesis to explain these observations is that the mild phenotype observed in these patients is due to the disruption of dimer stability and/or function by the elongated TCF4 isoform, in a manner that depends on dimerization partners and/or genomic context. An alternative explanation is that the frameshift mutation leads to protein degradation. In support of this hypothesis, another frameshift mutation, S653Lfs*57, has been shown to lead to TCF4 degradation, aggregation, and subsequent impaired DNA binding to the E-box^23^.

### Molecular pathology in PTHS: *TCF4* haploinsufficiency or dominant-negative effect?

Most *TCF4* mutations carried by patients with PTHS result in an obvious haploinsufficiency state, as translocations, deletions, or nonsense and frameshift mutations limit the production of certain or all isoforms to only one allele^23^. On the other hand, the resulting effect of missense mutations is less obvious. One possibility is that missense mutations in the basic region of the HLH domain impair or abolish TCF4 DNA binding. Alternatively, missense mutations elsewhere in the HLH domain may affect TCF4 dimerization, resulting in unstable or absent HLH dimers involving TCF4^23,24^. In addition, missense mutations affecting AD2 and Rep may disturb the function of these domains or perturb TCF4 dimer stability depending on dimerization partner^23^. For all these possibilities, the net functional result could be considered hypomorphic or loss-of-function.

The fact that some missense mutations perturb or abolish TCF4-mediated transcriptional regulation without affecting dimerization ability in vitro^23^ suggests that the aberrant TCF4 protein may sequester its molecular partners, resulting in a hypomorphic or dominant-negative effect. However, it is reasonable to speculate that a hypomorphic or dominant-negative effect would be very mild or not happen in vivo due to the diminished stability of dimers with a mutant TCF4^24^. Indeed, there is some evidence indicating that the half-life of HLH proteins relies to some extent on dimerization, as protein degradation can be prevented by dimer stabilization in the nucleus through DNA binding and interaction with transcriptional co-factors^58,74^.

If some missense mutations result in a significant hypomorphic or dominant-negative effect rather than a haploinsufficiency state in vivo should be further investigated. However, there is no doubt that PTHS results from loss of TCF4-mediated transcriptional regulation. How such dysregulation triggers PTHS pathophysiology is still unclear, but it seems to involve the general role of E-proteins as cell-cycle regulators and the specific role of TCF4 in cellular differentiation—a topic explored in the following section.

## TCF4 function

### Transcriptional regulation by TCF4

The binding of HLH dimers containing E-proteins to specific promoters and/or enhancers depends on the composition of the dimer itself and on E-box internal and flanking DNA sequences, as each dimer member has higher affinity for E-boxes flanked by particular sequences^75–77^. Furthermore, affinity of one dimer for a promoter and/or enhancer may depend on homotypic cooperativeness with preexisting dimers or collective binding with transcriptional co-factors^78^. Finally, E-protein DNA-binding specificity is regulated by Ca^2+^-dependent proteins such as calmodulin and S100, which interact directly with the basic region of the HLH domain^79,80^. In the presence of Ca^2+^, calmodulin exhibits low affinity for HLH heterodimers, preferentially inhibiting the DNA binding activity of E-protein homodimers. Indeed, cellular manipulation of calmodulin or Ca^2+^ levels has a direct effect on E-protein–mediated transcription^81^.

E-protein function is usually understood under the paradigm that tissue-specific class II HLH proteins dimerize with the widely expressed E-proteins to regulate specific gene networks that determine lineage commitment and cellular differentiation^82,83^. Particularly, TCF4 has been associated with the regulation of hematopoiesis^84^, myogenesis^85^, neurogenesis^86^, melanogenesis^87^, and osteogenesis^88^, as well as the differentiation of endothelial^89^, mammary gland^90^, placental^91^, and Sertoli cells^67^.

Differentiation programs activated by HLH proteins are usually coordinated with cell-cycle exit through E-protein–mediated transcriptional activation of cyclin-dependent kinase inhibitors (CDKIs)^92,93^. Class V HLH proteins act in opposition to this process; these proteins, called inhibitors of DNA binding (ID), indirectly promote cell cycle through inhibition of E-proteins and, consequently, inhibition of CDKI expression^83,94^. Therefore, cycle withdrawal or promotion may rely on the stoichiometric excess of either ID or E-proteins in order to favor the prevalence of bHLH/ID or bHLH/bHLH dimers, respectively, but the regulation of the equilibrium between E-protein and ID protein levels is not completely understood and possibly varies according to cell type.

Considering the dynamics of E-protein dimer affinity for distinct E-box sequences, discovering TCF4 target genes is not trivial. Initially, putative targets were uncovered by demonstrating that TCF4 interacted with promoter/enhancer sequences of particular candidate genes (via cDNA library screening or DNA-binding competition assays)—such as the immunoglobulin heavy and light chain enhancers^16^ and promoters of genes encoding thyroglobulin^95^, tyrosine hydroxylase^96^, somatostatin receptor II^97,98^, fibroblast growth factor 1^65^, and Purkinje cell protein 2^99^. In addition, TCF4-mediated regulation was investigated using luciferase reporter assays, exemplified by the study of promoters for CDKIs^92^, and NRXN1β and CNTNAP2^24^. Interestingly, *NRXN1β* and *CNTNAP2* have been shown to cause PTHS-like autosomal recessive intellectual disability disorders^100^, both of which manifest motor and speech delay, stereotypical and intense repetitive movements, and tachypnea and/or apnea, but without the PTHS characteristic facial *gestalt*. Finally, it should be noted that the molecular approaches described above did not provide a full understanding of TCF4-mediated transcriptional regulation because most experiments were conducted with exogenous transgenes and reporter cassettes and did not take into account activity of the endogenous *TCF4* locus.

Although microarray and next-generation RNA sequencing experiments alone cannot directly indicate TCF4 target genes, they can reveal a large-scale picture of the gene networks influenced by TCF4. For example, microarray analysis after *TCF4* knockdown in the human neuroblastoma cell line SH-SY5Y revealed differentially expressed genes mostly involved in cell survival, epithelial-to-mesenchymal transition, and neuronal differentiation^101^.

Moreover, RT-qPCR with mRNA enriched via purification of translating ribosomes from neurons of the rat medial prefrontal cortex (mPFC) revealed that, relative to the majority of known ion channel genes in the rat genome, KCNQ1 and SCN10a are substantially overexpressed after *TCF4* knockdown^102^. Other studies used a combination of omics approaches in human neuronal cell lines to reveal that several TCF4 target pathways are involved in cell survival, cell-cycle regulation, neurogenesis, and neuronal lineage commitment^103–106^, although evidence of direct TCF4 target genes obtained through credible CHIP-Seq experiments in different cell types is still missing.

### Roles of TCF4 during neural progenitor cell maintenance and differentiation

Despite some limitations, the studies described above point to a critical role of TCF4 in nervous system physiology and development. *TCF4* expression in the brain increases considerably at the end of prenatal life and decreases at early infancy to basal levels that persist through adulthood^107^. *TCF4* is expressed in cortical and subcortical regions of the developing and adult brain, prominently in the cortex, hippocampus, and hypothalamic and amygdaloid nuclei, a pattern that is highly similar between humans and rodents^1,102,108^.

It is well known that HLH proteins exert critical roles during neural progenitor cell (NPC) maintenance and/or differentiation into neurons, oligodendrocytes, and astrocytes^109^. Repressor HLH proteins such as HES and ID regulate NPC auto-renovation, ensuring maintenance of the population and formation of an appropriate number of neurons and glia throughout development^109^. During the neurogenic phase, proneural HLH proteins coordinate not only a generic neuronal identity but also specific neuronal subtypes. Generally, NEUROG1 and NEUROG2 expression is restricted to the dorsal telencephalon, which originates glutamatergic neurons, and ASCL1 is predominantly expressed in the ventral telencephalon, which originates GABAergic neurons^109^. During the gliogenic phase, OLIG1 and OLIG2 expression regulates NPC differentiation into oligodendrocytes, and further HES and ID expression regulates NPC differentiation into astrocytes^109^. There is evidence that TCF4 is able to dimerize with these HLH proteins^15,18–24^, but results showing that TCF4 dimers regulate neurogenic and gliogenic processes during brain development are still lacking.

TCF4 has high affinity for Mediator—a multiprotein complex that regulates transcription by connecting enhancers to promoters^110^. In NPCs, TCF4 recruits Mediator to define most super-enhancers—regions of high-density Mediator binding responsible for maintaining cell identity and viability through regulation of lineage-specific and cell-cycle genes^110^. Many such genes regulated by TCF4 encode neurogenic HLH and other proteins that interact and colocalize with TCF4 at super-enhancers, including in the *TCF4* gene itself^21,110^. Indeed, TCF4 seems to regulate its own expression in NPCs, as TCF4, Mediator and epigenetic marks that promote gene expression are found near transcriptional start sites for *TCF4*’s shorter isoforms^110^. This suggests that a positive feedback loop maintains expression of those transcription factors, resulting in maintenance of the NPC population.

*TCF4* levels increase considerably during neurogenesis, and differentiation is promoted by a combination of factors that induce the expression of TCF4 longer isoforms, mainly TCF4-B, through the canonical WNT/β-catenin pathway and imprinted transcription factor ZAC1^104,111^.

### Role of TCF4 during cortical development

In mice, TCF3—another E-protein—acts by regulating the expression of TCF4-B at early stages of neurogenesis in the mouse dorsal telencephalon, specifically at embryonic day 12 (E12), as either *Tcf3* or *Tcf4* loss-of-function results in an increased population of intermediate progenitors in the ventricular and subventricular zones^112^. However, even though TCF3 does not influence TCF4 levels at later stages of neurogenesis, both factors continue to influence the differentiation in a global manner, as *Tcf3* or *Tcf4* gain- or loss-of-function results in decrease or increase in the population of mouse radial glia cells at postnatal day 2 (P2), respectively^86^. Therefore, TCF4 regulates both NPC maintenance and neurogenesis during mouse telencephalon development. It is possible that regulation of *Tcf4* expression is context-dependent, with different pathways acting in different regions of the developing telencephalon, but such possibility still needs to be further investigated.

Importantly, *Tcf4* loss-of-function in mice is accompanied by deficits in cortex development and cortical layer structure: *Tcf4*^−/−^ mice display aberrant numbers of different sub-types of cortical neurons, including those expressing SATB2 and BRN2 markers, but these alterations are surprisingly milder in *Tcf4*^+/−^ animals^112^.

Besides regulating NPC maintenance and neurogenesis, TCF4 also acts in neuronal migration and neurite formation during telencephalon development. *Tcf4*^+/−^ mice show an increased number of neurons “stuck” in deeper layers of the telencephalon instead of migrating to the cortical plate^112^. Also, *Tcf4* knockdown in the mouse telencephalon at E14.5 increases the number of neurons “stuck” in the ventricular and intermediate zones^113^. Curiously, knockdown of *Bmp7*—a gene negatively regulated by TCF4 that codes for a TGF-β receptor ligand—partially rescues normal neuronal migration in these mice. *Tcf4* knockdown in the developing mouse telencephalon also disturbs the formation of the neuronal leading process, which could explain the disruption in neuronal migration^113^.

Conversely, increasing levels of TCF4-B in the developing rodent telencephalon increases the rate of neuronal migration^112^ and results in aggregation of pyramidal neurons in the mPFC but not in other areas of the neocortex^114^, a finding that may be of significance in the context of trying to understand the causality between *TCF4* polymorphisms and SCZ. Such abnormal aggregation was associated with neuronal activity mediated by Ca^2+^ influx as a result of NMDA receptor activation, and normal distribution of pyramidal neurons could be rescued by increasing levels of calmodulin, thereby inhibiting TCF4-mediated transcriptional activity^114^. Measures of cellular electrophysiology and spontaneous Ca^2+^ transients revealed that TCF4-B gain-of-function increases neuronal excitability, indicating that TCF4-mediated transcription regulates spontaneous neuronal activity in the developing neocortex and potentially increases NMDA receptor function^114^.

### Role of TCF4 beyond cortical development

Recently, Wang et al. investigated the potential roles of TCF4 during mouse hippocampus development^108^. Conditional knockout of *Tcf4* in NPCs of the dentate neuroepithelium resulted in a drastically reduced hippocampus that persisted through adulthood. Particularly, the size of the dentate gyrus was dramatically reduced. During dentate gyrus development, radially migrating NPCs form a migratory stream from the neuroepithelium toward the hilus of the dentate gyrus, where they undergo reorganization to build the neurogenic subgranular zone^115^. *Tcf4* knockout disturbed NPC migration, as these cells were “stuck” in the dentate migratory stream at P0 instead of migrating to the hilus, thus disrupting subgranular zone formation^108^. Interestingly, the disturbance in migration is due to disorganized radial glia scaffold from the neuroepithelium along the dentate migratory stream and to reduced expression of *Wnt7b*^108^—a TCF4 target known to be responsible for neurite development through the noncanonical Wnt pathway. Not surprisingly, such migration defects lead to altered social behavior and memory performance in adult mice^108^.

Furthermore, it has been recently shown that *Tcf4*^−/−^ mice exhibit corpus callosum and anterior commissure malformation, which are detected at E17.5 and persist up to P0^116^. It is noteworthy, however, that heterozygous animals seem to be unaffected. Since the formation of midline glial structures is essential for commissural formation^117^, the authors suggest that loss of midline glia may be responsible for the observed defects in *Tcf4*^−/−^ mice.

The role of TCF4 in the developing brain is not restricted to the telencephalon. TCF4 dimerization with ATOH1 is critical for normal pontine nuclei development. *Tcf4*^−/−^ mice and double-heterozygote *Tcf4*^+/−^/*Atoh1*^+/^^−^ mice show extensively reduced pontine nuclei due to abnormal migration of neurons in the dorsolateral rhombencephalon^118^. Curiously, such abnormal migration is restricted to pontine nuclei, even though *Tcf4* and *Atoh1* are both expressed throughout the rhombic lip.

Altogether, these findings suggest that TCF4 has specialized roles in different populations of NPCs throughout the developing brain.

### Role of TCF4 in post-mitotic neuronal function

TCF4 function also seems to be intimately associated with neuronal activity. In rat primary neurons, transcription mediated by any TCF4 isoform is dependent on depolarization^39^. Synaptic activity triggers Ca^2+^ influx through NMDA receptors and type L voltage-gated Ca^2+^ channels, thereby activating protein kinase A, which then phosphorylates TCF4 at serine residues 448 and 464 (between AD2 and Rep). Such phosphorylation is necessary for transcriptional activity, as mutations affecting these sites are sufficient to prevent the abnormal distribution of pyramidal neurons in the rat mPFC resulting from TCF4-B gain-of-function^39^. Furthermore, *Tcf4* knockdown in neurons of the rat mPFC at E16 attenuates neuronal excitability by increasing the expression of the *Kcnq1* and *Scn10a* genes, both of which code for ion channels mostly expressed in the peripheral nervous system and responsible for regulating action potential firing rate^102^. In addition, *Tcf4* knockdown in post-mitotic interneurons of the adult mouse olfactory bulb increases dendrite number and length; conversely, *Tcf4* overexpression decreases dendrite number and length^119^.

### Role of TCF4 in other neural cell types

The studies listed in the preceding sections made it clear that TCF4 function is associated with neuronal activity and regulation of different aspects of neural development. However, Phan et al.^120^ recently demonstrated that TCF4 function is also associated with oligodendrocyte development and myelination. By assessing molecular convergence across five independent mouse models of PTHS with distinct heterozygous mutations, the authors found upregulation of genes associated with neuronal function and downregulation of genes associated with oligodendrocytes and myelination in neonate and adult transcriptomes from several brain regions. They observed decreased numbers of mature oligodendrocytes and more oligodendrocyte progenitor cells, decreased proportion of myelinated axons in the *corpus callosum*, and increased proportion of neuronal activity being transmitted down unmyelinated axons in *Tcf4*^+/−^ mice^120^. In order to rule out the possibility that *Tcf4* mutations affect oligodendrocytes in a non–cell-autonomous manner, the authors induced differentiation of cultured primary oligodendrocyte progenitor cells dissociated and purified from neonatal mice, as well as deleted a single *Tcf4* allele in the oligodendrocyte lineage by crossing *Olig2-Cre*^+/−^ mice with “floxed” *Tcf4*^fl/+^ mice. Both approaches revealed increased proportion of oligodendrocyte progenitor cells and reduced proportion of mature oligodendrocytes as a result of the *Tcf4* mutation^120^, thus confirming that TCF4 regulates oligodendrocyte progenitor cell differentiation and/or survival in a cell-autonomous manner.

In addition, Wedel et al.^121^ recently showed that TCF4 is required for terminal differentiation in the oligodendrocyte lineage. In genetically modified prenatal mice without *Tcf4* transcripts encoding longer isoforms, the authors found that oligodendroglial cells are arrested in the pre-myelinating stage. Furthermore, they observed severely delayed myelination in ex vivo organotypic slice cultures, showing that such effect is cell-autonomous. Notably, TCF4 was shown to genetically interact with OLIG2 for terminal oligodendrocyte differentiation^121^: double-heterozygote *Tcf4*^+/−^/*Olig2*^+/−^ mice exhibited significant reduction in the number of differentiating oligodendrocytes at E18.5. Such findings suggest that part of the functional deficits in patients with PTHS may be caused by altered oligodendrocyte function and myelination.

## Current knowledge on aberrant functions of TCF4 in disease

It is noteworthy that several *Tcf4*^+/−^ mouse models, carrying distinct types of *Tcf4* mutation in heterozygosity, exhibit mildly aberrant phenotypes, including subtle changes in cerebral cortex cellular composition^112^, hippocampus development^108,116^, and electrophysiological properties of neurons^122^. It is unclear whether such set of abnormalities in heterozygous mice closely resembles the phenotypes found in children with PTHS carrying similar *TCF4* mutations. An alternative interpretation is that the mild phenotypes observed in *Tcf4*^+/−^ mice do not correspond to human phenotypes and that these dissimilarities result from differences between rodent and human brain composition and development, which are substantial and should not be ignored.

Nevertheless, some *Tcf4*^+/−^ mice harbor clinically relevant genetic variants, such as missense mutations affecting the arginine residues of the basic region of the HLH domain or deletions in exon 19, which codes for the HLH domain^122,123^. Assessment of behavior and electrophysiology in these *Tcf4*^+/−^ mice showed that they can partially model the PTHS phenotype. Notably, Kennedy et al.^123^ first reported that *Tcf4*^+/−^ mice (harboring a deleted exon 19) exhibit alterations in balance and motor coordination, preference for social isolation over interaction, and repetitive behaviors represented by increased grooming. These mice also show dysregulation in sensorimotor gating, as adults are hyper-responsive and have significant deficits in prepulse inhibition^123^. Curiously, communication deficits were also observed in the form of significantly reduced ultrasonic vocalizations and weaker ultrasonic distress calls in *Tcf4*^+/−^ pups.

Multiple *Tcf4*^+/−^ mouse models present hippocampus-dependent cognitive deficits, which were assessed through behavioral tasks of spatial and associative learning and memory^122,123^. Interestingly, such deficits were shown to be coupled with enhanced hippocampal long-term potentiation (LTP) at Schaffer collaterals between CA3 and CA1, which is seemingly driven by NMDA receptor hyperfunction. In addition, next-generation sequencing experiments were performed on hippocampal CA1 tissue from wild-type and *Tcf4*^+/−^ mice^123^ and the authors found significant dysregulation in pathways associated with neuronal plasticity, axon guidance, memory-associated genes, as well as significant demethylation in upregulated genes associated with these pathways.

Considering the role of TCF4 in the regulation of histone acetylation states through the activity of either AD1 and/or AD2, Kennedy et al. also assayed whether histone deacetylase (HDAC) inhibition would be sufficient to normalize the enhanced hippocampal LTP phenotype of *Tcf4*^+/−^ mice^123^. Surprisingly, treatment with the HDAC inhibitor trichostatin A significantly reduced LTP in the *Tcf4*^+/−^ mouse hippocampus. Moreover, *Hdac2* knockdown and subchronic treatment with HDAC inhibitor suberoylanilide hydroxamic acid (SAHA) were sufficient to improve learning and memory in *Tcf4*^+/−^ mice, thus indicating that cognition in PTHS model mice can be improved by HDAC inhibitors through normalization of synaptic plasticity.

## Concluding remarks

It is now clear that *Tcf4*^*+/–*^ mice display some aberrant behaviors, which may reflect the pathological findings reported over the last few years regarding the role of TCF4 in brain development and function. Such findings are consistent with some cognitive and motor dysregulation found in children with PTHS, but further work is required to determine if the complete set of phenotypes in mouse models closely mimics the aberrant phenotypes in patients. Moreover, it is still unclear the extent to which the underlying pathophysiology is due to disruption in TCF4 function during brain development *versus* disruption in post-mitotic cells in the fully formed nervous system.

Considering that TCF4 seems to have specialized roles in different populations of NPCs, it is possible that its loss-of-function affects differentiation of discrete neuronal populations in certain regions of the brain, thus causing a wide variety of symptoms. Indeed, there are cell type–specific and region-specific differences in *TCF4* expression^1^, suggesting that different subpopulations require different doses of TCF4 and are possibly differently vulnerable to *TCF4* pathological alterations. The use of patient-derived cellular models in vitro might help unravel some of these aberrant phenotypes and provide a detailed understanding of the underlying pathophysiology related to *TCF4*.

In summary, TCF4 remains an elusive transcription factor. The precise molecular mechanisms through which *TCF4* mutations contribute to PTHS pathophysiology, as well as the role of *TCF4* variants in other psychiatric disorders such as SCZ, remain to be further elucidated. Additional exploration of the dynamic expression and function of the *TCF4* gene throughout development together with the interplay between multiple TCF4 isoforms and different interacting partners is needed. TCF4 target genes are mostly unknown and evidence of in vivo *TCF4* dimer function is currently lacking; therefore, further investigation on the role of TCF4-containing heterodimers in lineage commitment and differentiation is required, which could be undertaken through experiments that explore TCF4’s relationship with known neurogenic and oligogenic HLH transcription factors.

Although much is yet to be comprehended, the general role of *TCF4* in psychiatric disease has clearly emerged, materializing TCF4 as a key regulator of neural function, including learning, memory, language, and sociability.
